# Alternative Arterial Access in Veno-Arterial ECMO: The Role of the Axillary Artery

**DOI:** 10.3390/jcm14155413

**Published:** 2025-08-01

**Authors:** Debora Emanuela Torre, Carmelo Pirri

**Affiliations:** 1 Department of Cardiac Anesthesia and Intensive Care Unit, Cardiac Surgery, Ospedale dell’Angelo, 30174 Venice Mestre, Italy; deboraemanuela.torre@aulss3.veneto.it; 2 Department of Neurosciences, Institute of Human Anatomy, University of Padova, 35121 Padova, Italy

**Keywords:** veno-arterial ECMO, axillary artery cannulation, femoro-axillary configuration, cannulation strategy, extracorporeal life support, ECMO cannulation, axillary ECMO, femoro-axillary ECMO

## Abstract

**Background:** Veno-arterial extracorporeal membrane oxygenation (V-A ECMO) is increasingly used to support patients with refractory cardiogenic shock or cardiac arrest. While femoral artery cannulation remains the most common arterial access, axillary artery cannulation has emerged as a valuable alternative in selected cases. **Objective**: This narrative review aims to synthesize current evidence and expert opinion on axillary artery cannulation in V-A ECMO, focusing on its technical feasibility, physiologic implications, and clinical outcomes. **Methods**: A comprehensive literature search was performed in PubMed and Scopus using relevant keywords related to ECMO, axillary artery, cannulation techniques, and outcomes. Emphasis was placed on prospective and retrospective clinical studies, expert consensus statements, and technical reports published over the past two decades. **Results**: Axillary cannulation provides antegrade aortic flow, potentially reducing the risk of differential hypoxia and improving upper body perfusion. However, the technique presents unique technical challenges and may carry risks such as hyperperfusion syndrome or arterial complications. Emerging data suggest favorable outcomes in selected patient populations when performed in experienced centers. **Conclusions:** Axillary cannulation represents a promising arterial access route in V-A ECMO, particularly in cases with contraindications to femoral cannulation or when upper-body perfusion is a concern. Further prospective studies are needed to better define patient selection criteria and long-term outcomes.

## 1. Introduction

Extracorporeal membrane oxygenation (ECMO) represents an advanced adaptation of cardiopulmonary bypass, designed to ensure sufficient systemic oxygen delivery in patients experiencing severe respiratory and/or cardiac compromise. The two principal ECMO configurations are veno-arterial (V-A) and veno-venous (V-V) modalities. V-A ECMO is predominantly employed in the setting of hemodynamic collapse or profound cardiogenic shock, functioning by diverting venous blood, typically from the right atrium or a central vein, through an extracorporeal oxygenator before reinfusing it into the arterial circulation. This strategy effectively bypasses both the native cardiac and pulmonary circuits, thereby offloading myocardial work and ensuring end-organ perfusion [1]. V-A ECMO is frequently selected for acute refractory cardiogenic shock. Etiologies include acute myocardial infarction, post-cardiotomy failure, primary graft dysfunction following transplantation, fulminant myocarditis, and arrhythmia-induced decompensation, as well as sepsis-related myocardial dysfunction, drug toxicity, and catecholamine-induced crises [2,3]. V-A ECMO enhances systemic oxygen delivery while concurrently reducing myocardial metabolic demand [1]. By providing immediate circulatory and respiratory support, V-A ECMO can stabilize critically ill patients and serve as a bridge to recovery, durable ventricular assist device or cardiac transplantation.

Cannulation strategies are tailored to individual anatomical and clinical considerations. Arterial cannulae typically range from 15 to 21 Fr and venous from 19 to 25 Fr, allowing flows in excess of 6 L/min. A central, peripheral, or hybrid approach can be utilized, with radiographic confirmation of cannula placement recommended immediately post-implantation [4]. Traditionally, the femoral artery has been the most common access site due to its rapid accessibility and ease of cannulation [5]. However, retrograde aortic perfusion associated with femoral access may lead to significant drawbacks, including increased risk of lower limb ischemia [6], vascular complications [6], and, notably, differential hypoxia, especially in patients with severely compromised native pulmonary function and residual left ventricular ejection [7]. In this context, axillary artery cannulation has gained increasing attention as a physiologically advantageous alternative. The axillary approach allows for antegrade aortic perfusion, potentially reducing cerebral hypoxia and improving upper body oxygenation. Moreover, it facilitates early patient mobilization and may carry a lower risk of certain vascular complications. Nevertheless, this procedure is technically demanding and not without its own set of limitations and risks [8]. This narrative review aims to provide a comprehensive overview of axillary artery cannulation in V-A ECMO, focusing on its anatomical considerations, technical aspects, clinical indications, hemodynamic consequences, and complication profile.

## 2. Materials and Methods

This narrative review with a structured methodology is based on a comprehensive literature search conducted in the PubMed and Scopus databases, focusing on articles published between 1974 and 2025. The search strategy employed specific keywords such as “axillary cannulation ECMO” and “arterial cannulation ECMO”. Only English-language publications were considered, including original research articles, clinical series, and review papers selected for their scientific and clinical relevance. The initial search identified 2671 articles. After screening titles and abstract, 1251 studies were considered potentially eligible. Following full text review, 112 studies were included based on methodological quality and relevance to the review’s aim. Studies were selected if they provided data on axillary artery cannulation for V-A ECMO, included comparative analyses of arterial access strategies; offered clinically significant insights into cannulation related complications, management strategies or hemodynamic considerations relevant to axillary approach, even if axillary cannulation was not the primary focus. Articles were excluded if they were non-English, conference abstracts without full text, studies lacking data on outcomes or procedures, animal studies or papers focusing exclusively on unrelated cannulation strategies without clinical or conceptual relevance to axillary access. Since this is a narrative review with a structured search strategy but not a systematic review, a PRISMA flow diagram was not included. Nevertheless, key methodological steps, such as inclusion criteria, exclusion criteria, and article selection strategy, are clearly outlined to ensure reproducibility and transparency.

## 3. Results

### 3.1. Axillary Artery Anatomy and Surgical Access

**Figure 1:**
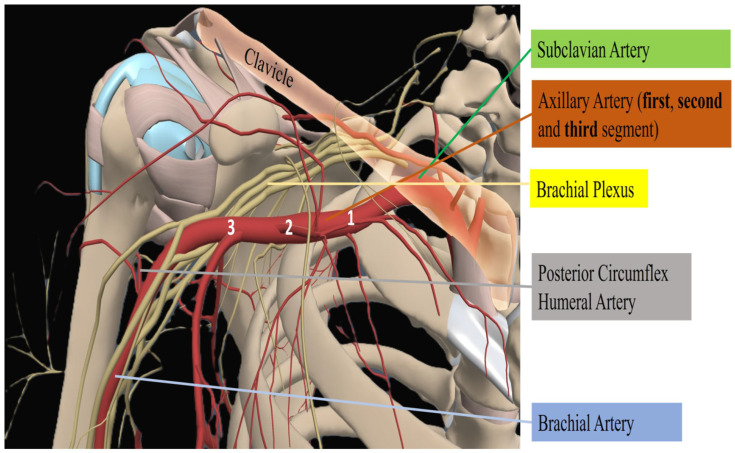
The axillary artery is divided into three segments relative to the pectoralis minor; arterial cannulation for V-A ECMO is usually performed on the third segment. Caution is required to avoid injury to nearby structures such as the brachial plexus, subclavian vein, and posterior circumflex humeral artery. 1: first segment of axillary artery; 2: second segment of axillary artery; 3: third segment of axillary artery. Figure author-generated.

The axillary artery, a continuation of the subclavian artery beyond the lateral margin of the fist rib, represents a relatively superficial and accessible vessel that supplies the upper limb. Its position beneath the pectoralis minor muscle and proximity to neurovascular structures, including the brachial plexus and axillary vein, requires precise anatomical understanding and meticulous surgical techniques [9,10]. For descriptive purposes, the axillary artery is divided into three parts based on its relationship to the pectoralis minor muscle: the first part lies proximal (medial to the muscle); the second part lies posterior (deep) to it; the third part lies distal (lateral) to the muscle [11]. From a surgical standpoint, the third portion of the axillary artery, particularly its distal segment, is most commonly utilized for arterial cannulation in peripheral veno-arterial ECMO (Figure 1). Precise knowledge of local vascular anatomy, including variations in branching pattern and the close proximity to neurovascular structures, is critical to minimize complications during cannulation. The posterior circumflex humeral artery and the axillary vein, which courses anteromedially and often overlaps the artery, represent important anatomical landmarks and potential hazards. The third segment of the axillary artery is often favored for arterial cannulation due to several anatomical and practical advantages. Surgically, this segment lies distal to the inferior border of the pectoralis major and latissimus dorsi muscles, thereby facilitating easier exposure through an infra-axillary or deltopectoral incision. This approach minimizes tissue dissection and allows for more direct access to the vessel [11]. From an anatomical perspective, this segment follows a relatively superficial and stable course, reducing the likelihood of displacement during cannulation and subsequent ECMO support. This stability is particularly advantageous in prolonged circulatory assistance, where cannula migration may compromise flow dynamics or vascular integrity. Furthermore, this distal segment is anatomically remote from the major trunks of the brachial plexus, which arise more proximally, thereby minimizing the risk of iatrogenic nerve injury and enhancing neurological safety [12]. Although both axillary arteries can be used for ECMO cannulation, the right axillary artery is generally preferred, due to its anatomical, surgical and hemodynamic advantages. Anatomically, the right artery arises from brachiocephalic trunk, providing a shorter and more linear trajectory for cannulation. Surgically, the right axillary region offers a more favorable operative field, characterized by more consistent neurovascular anatomy and lower risk of iatrogenic injury, including avoidance of the thoracic duct, which is present on the left side. Dissection on the right is typically less complex and safer. From a hemodynamic perspective, right-sided cannulation allows for more physiologic alignment with the right common carotid and vertebral arteries, thereby supporting robust antegrade cerebral perfusion [11,13]. Moreover, right axillary access is particularly advantageous when left-sided arterial access is needed for other procedures, such as TAVI [14]. Nonetheless, left axillary cannulation remains a viable and appropriate option, especially when the right side is occupied, for instance, by an axillary Impella device (which can be placed on either side with no significant difference in clinical outcomes), or otherwise unsuitable for cannulation [15].

#### 3.1.1. Surgical Technique for Axillary Artery Cannulation in V-A ECMO

**Figure 2:**
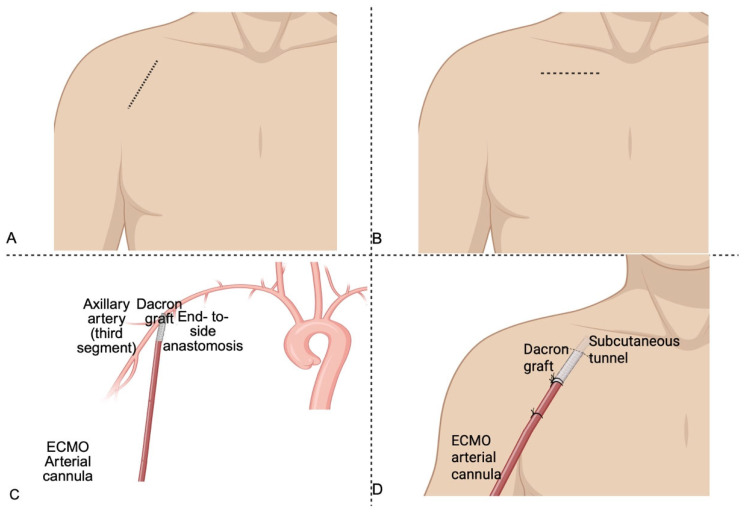
(**A**) Delto-pectoral incision following the natural groove between the deltoid (laterally) and the pectoralis major (medially), extending from the coracoid process toward the axillary fold. (**B**) Infraclavicular incision performed 1–2 cm inferior to the clavicle, parallel to its long axis, enabling exposure of the axillary artery beneath the clavicular head of the pectoralis major. (**C**) Surgical axillary cannulation with an 8 mm Dacron graft anastomosed end to side to the axillary artery. (**D**) Arterial cannula tunneled subcutaneously and connected to a Dacron graft anastomosed to the axillary artery. Created in BioRender 201.

The surgical approach to axillary cannulation is most commonly performed via a infraclavicular or deltopectoral incision, allowing exposure of the third segment of the axillary artery (Figure 2). This segment is favored for its superficial location and safe distance from major neural structures [16]. After systemic heparinization, the artery is usually encircled with vessel loops, and an 8–10 mm Dacron graft is anastomosed to the artery in an end-to-side fashion using a continuous 5-0 or 6-0 polypropylene suture. Following de-airing, the graft is tunneled subcutaneously and connected to the arterial line on the ECMO circuit using a Y-connector. This technique enables antegrade perfusion to the aortic arch and cerebral circulation, while preserving perfusion to the ipsilateral limb [17,18]. Direct cannulation is generally avoided to prevent limb ischemia [19]. Preoperative imaging should be considered imperative in several scenarios, including elective surgical axillary access, particularly in patients with prior vascular interventions, known or suspected subclavian artery disease, previous coronary artery bypass grafting involving the internal mammary artery or suspected aortic arch abnormalities such as coarctation. Nonetheless, routine use of pre-procedural imaging, particularly duplex ultrasound, given its wide availability, bedside applicability, and absence of ionizing radiation, would enhance procedural safety and vascular assessment in all cases, not just those deemed to be high-risk.

#### 3.1.2. Percutaneous Technique for Axillary Artery Cannulation in V-A ECMO

**Figure 3:**
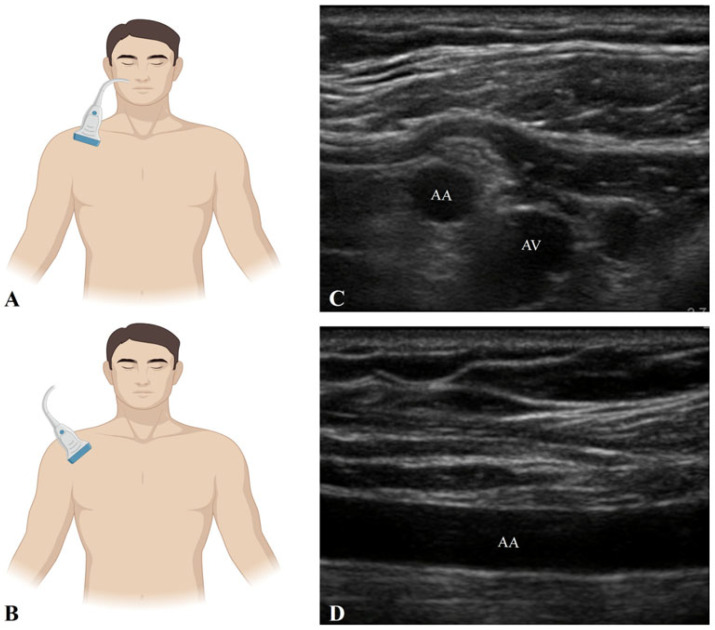
Sonographic assessment of the axillary artery. (**A**) Linear probe positioned transversely, perpendicular to the axis of the upper limb. (**B**) Linear probe positioned longitudinally, parallel to the axis of the upper limb. (**C**) Out-of-plane view of the axillary artery and axillary vein. (**D**) In-plane view of the axillary artery. AA: Axillary artery; AV: Axillary vein. Figure Author generated using BioRender 201.

The percutaneous technique involves ultrasound-guided cannulation of the axillary artery, generally via Seldinger method. Percutaneous axillary cannulation can also be performed under fluoroscopic or ultrasound guidance in interventional cardiology settings, particularly when combined with diagnostic or therapeutic procedures. The artery is punctured directly, most commonly at the level of the third portion, using real-time sonographic or fluoroscopic guidance to avoid adjacent neural structures. To accurately identify the axillary artery sonographically, a systematic ultrasound examination is conducted with the patient in a supine position, typically with the arm abducted and slightly externally rotated to optimize access. It is important to assess the axillary vessels on the cannulated side using both in-plane (longitudinal) and out-of-plane (transverse) ultrasound views. For in-plane cannulation, a high-frequency linear transducer (10–15 MHz) is positioned longitudinally just inferior to the clavicle, in the region adjacent to the first rib, parallel to the axis of the upper limb. For the out-of-plane view, the probe is placed perpendicular to the axis of the upper limb. Using B-mode imaging, the operator identifies a pulsatile, linear (in-line view) or round (out-of-line view), echogenic structure with distinct arterial wall layers. Color Doppler is employed to confirm intraluminal blood flow, showing pulsatile characteristic of arterial circulation. The artery appears as an anechoic or hypoechoic structure with hyperechoic walls. To distinguish it from the adjacent axillary vein, gentle manual compression is applied; the vein typically collapses, while the artery remains pulsatile and non-compressible [14,20], (Figure 3).

**Figure 4:**
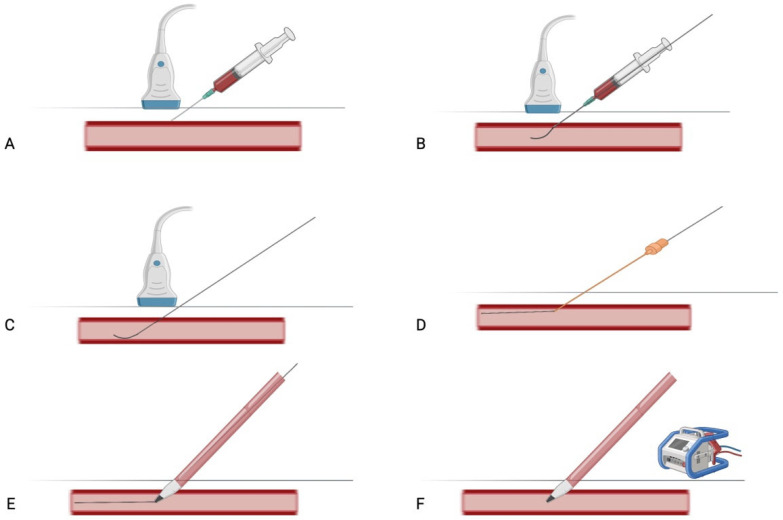
Stepwise illustration of percutaneous axillary artery cannulation. (**A**) Ultrasound-guided arterial puncture. (**B**) Insertion of the guidewire using the Seldinger technique. (**C**) Syringe withdrawal, with the Seldinger guidewire left in place within the arterial lumen. (**D**) Sequential dilation of the access tract over the guidewire. (**E**) Advancement and positioning of the arterial ECMO cannula. (**F**) Withdrawal of the Seldinger guidewire. Created in BioRender 201.

Once the artery is clearly identified and its course delineated, direct puncture is performed, most commonly at the level of the third portion. After arterial access is confirmed, a guidewire is advanced, followed by progressive dilatation of the soft tissue tract. A percutaneous arterial ECMO cannula (usually 15–19 Fr) is then introduced over the guidewire. When performed under fluoroscopy, the guidewire and cannula positioning can be continuously monitored to ensure correct placement. To minimize the risk of bleeding and arterial dissection, meticulous attention to wire positioning and cannula alignment is essential throughout the procedure [21], (Figure 4).

### 3.2. Overview of ECMO Cannulation Strategy

The femoro-axillary ECMO configuration constitutes a variant of peripheral cannulation. Veno-arterial ECMO can be implemented via peripheral or central approaches, depending on the clinical context [22].

#### 3.2.1. Central Cannulation in V-A ECMO

Central cannulation via median sternotomy (ascending aorta/right atrium) remains the gold standard for post-cardiotomy shock or when peripheral access is precluded. While it enables high-flow support and avoids differential hypoxia, its invasiveness increases risks of mediastinitis, bleeding, aortic dissection [23], delayed mobilization and sternal complications. Despite comparable survival outcomes to peripheral ECMO in meta-analyses [24], central access is generally reserved for select indications due to its morbidity profile. This has prompted growing interest in less invasive alternatives, such as axillary cannulation.

#### 3.2.2. Peripheral V-A ECMO. Femoral Cannulation, North–South Syndrome, and the Emerging Role of Axillary Access

**Figure 5:**
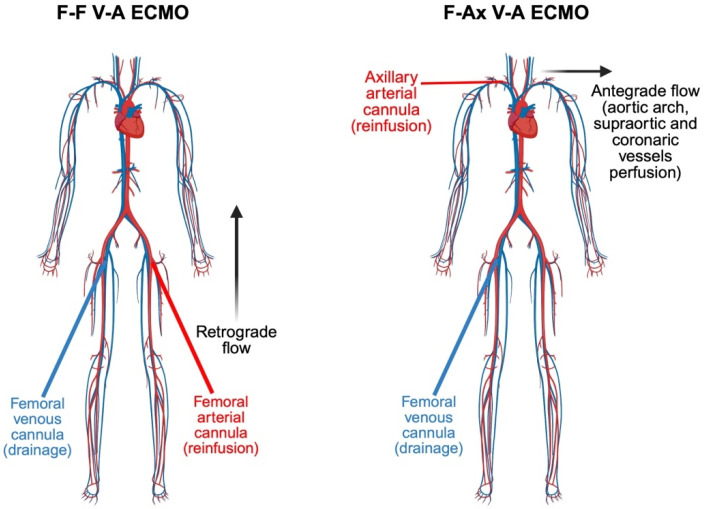
Comparison of arterial flow patterns in femoro-femoral (F-F) versus femoro-axillary (FAx) ECMO. In femoro-femoral (FF) ECMO, arterial flow is retrograde toward the aortic arch, increasing the risk of differential hypoxia. In femoro-axillary (F-Ax) ECMO, flow is anterograde, favoring cerebral and coronary perfusion. ECMO F-F: femoro-femoral ECMO; ECMO F-Ax: femoro-axillary ECMO. Created in BioRender 201.

In peripheral V-A ECMO, femoral artery cannulation remains the standard approach; however, it is associated with several limitation, including distal limb ischemia and retrograde flow related complications such as increased left ventricular afterload and consequent pulmonary edema [25,26]. A significant risk related to femoral access is North–South syndrome (NSS), also known as Harlequin syndrome, characterized by differential hypoxemia where the upper body receives desaturated blood due to recovering left ventricular function combined with impaired pulmonary oxygenation, while lower body receives well-oxygenated blood from ECMO [7,27,28]. Mitigation strategies for NSS include conversion to V-AV (veno-artero-venous) ECMO, which adds a second venous return cannula to deliver oxygenated blood directly into the right atrium or superior vena cava, thereby improving pulmonary arterial oxygen content and systemic oxygenation [29]. Axillary artery cannulation offers a physiologically favorable alternative to femoral access by delivering true antegrade systemic perfusion, which is particularly advantageous in preventing NSS. Unlike femoral cannulation, which directs retrograde flow into descending aorta, axillary cannulation provides oxygenated blood directly to the aortic arch and its supra-aortic branches [18], ensuring adequate oxygen delivery to the cerebral and coronary circulation and significantly reducing the risk of upper body hypoxia (Figure 5). This configuration mitigates cerebral desaturation caused when a recovering left ventricle ejects poorly oxygenated blood into the proximal aorta [30]. From a hemodynamic perspective, axillary cannulation promotes a more physiological integration between native cardiac output and extracorporeal flow by facilitating effective mixing at the level of the aortic arch rather than the descending aorta, as seen with femoral cannulation [31]. This synchronization reduces the risk of adverse retrograde flow dynamic that may impair end organ perfusion. Although axillary access does not fully alleviate the increase in left ventricular afterload inherent to peripheral V-A ECMO, the antegrade flow it provides results in comparatively lower afterload than femoral retrograde perfusion, which can exacerbate ventricular unloading impairment and predispose to aortic root stasis and thrombus formation [32,33]. By fostering, laminar, antegrade perfusion, axillary cannulation may contribute to improved myocardial recovery and decreased pulmonary congestion, particularly in patients with residual or recovering left ventricular function [34,35,36].

### 3.3. Axillary Artery Cannulation: An Evolving Strategy in Veno-Arterial ECMO

Axillary arterial cannulation has emerged as a pivotal and physiologically advantageous technique in contemporary extracorporeal life support, offering distinct advantages in specific clinical scenarios. Primary indications include V-A ECMO implantation for cardiogenic shock (particularly in cases with anticipated prolonged support), post-cardiotomy cardiac failure, and as preferred alternative when femoral access is contraindicated due to severe peripheral vascular disease or prior instrumentation. This approach is particularly advantageous in patients with significant peripheral arterial disease or when femoral cannulation is technically challenging, thereby mitigating complications such as limb ischemia, hemorrhage, vessel perforation, and suboptimal cannula sizing. Key clinical advantages include reduction in differential hypoxemia; facilitation of early mobilization, particularly in patients requiring prolonged V-A ECMO support; and streamlined transition to isolated left ventricular support when clinically indicated. Both pseudo-percutaneous approaches and prosthetic graft (e.g., “chimney graft”) anastomosed to the axillary artery provide secure cannulation, enable primary wound closure, and reduce infection risks. In contrast to lower limb cannulation, where ischemia is the most common vascular complication, axillary access is more frequently associated with ipsilateral upper limb hyper perfusion and edema, a consequence of unregulated extracorporeal arterial flow [34]. Moreover, the antegrade arterial flow achieved through axillary artery has been shown to enhance cerebral oxygenation when compared to the retrograde flow typically produced by femoral artery access [30].

#### 3.3.1. Clinical Evidence Supporting the Efficacy of Axillary Artery Cannulation

Supporting evidence (Table 1 and Table 2) includes a retrospective study by Ohira et al. (2020) [12] compared axillary versus femoral artery cannulation for V-A ECMO in patients with cardiogenic shock. Among 371 patients, axillary access was associated with significantly lower rates of limb ischemia, wound complications, and need for site conversion, without compromising survival or increasing bleeding or cerebrovascular events. These findings support axillary cannulation as a safe and advantageous alternative, especially in patients with peripheral vascular disease or post-transplant graft failure [12]. Radwan et al. [35] conducted a retrospective analysis of 179 post-cardiotomy patients who underwent V-A ECMO via right axillary artery cannulation, between 2014 and 2019. The study reported a successful weaning rate of 48.6%, with an in-hospital survival rate of 34.6% and a one-year survival rate of 74% among those weaned. Complications included subclavian bleeding (13.4%), upper limb ischemia (6.1%), intracerebral hemorrhage (5%), and stroke (10.6%). These findings suggest that right axillary artery cannulation is a safe and effective alternative for V-A ECMO support in patients with acute left ventricular dysfunction following cardiac surgery, offering acceptable complication rates. Pisani et al. [37] evaluated right axillary artery cannulation for V-A ECMO in 174 patients. The study demonstrated that this approach is feasible and associated with low rates of local complications (e.g., bleeding 4%, upper limb ischemia 1.1%, local infection 1.7%, brachial plexus injury 0.6%). Survival at one year reached 72.7% among successfully weaned patients. However, due to the lack of a control group with femoral cannulation, definitive conclusions regarding the superiority of axillary over femoral access cannot be drawn. Technical innovations further optimize outcomes. Hysi et al. [38] explored the safety and feasibility of direct axillary artery cannulation, performed without the interposition of a prosthetic graft, for arterial return in ECMO. In their cohort of 16 patients, this technique provided a reliable perfusion route with a low incidence of neurological and vascular complications. Notably, these findings stand in contrast to earlier reports, such as that by Cakici et al. [19], which suggested that graft interposition could reduce the risk of local complication, including thrombosis and anastomotic bleeding, when compared to direct cannulation. The result of Hysi et al. [38], therefore challenges the assumption that prosthetic grafting is intrinsically safer, and indicate that, in selected patients, direct cannulation may offer a simpler and equally safe alternative. Importantly, the axillary artery allows for antegrade flow, potentially reducing the risk of differential hypoxia. While the technique requires meticulous dissection and careful patient selection, these findings support the incorporation of axillary cannulation into the surgical armamentarium for ECMO, particularly in cases where femoral access is contraindicated or suboptimal.

#### 3.3.2. Femoro-Axillary vs. Femoro-Femoral Cannulation in V-A ECMO: A Comparative Insight into Hemodynamic and Clinical Outcomes

Navia et al. [46] described axillary artery cannulation as a viable strategy for V-A ECMO, offering antegrade central perfusion and facilitating sternal closure. Compared to femoral access, this technique may reduce the risk of differential hypoxia and improve surgical field management, especially in post-cardiotomy settings or patients with peripheral vascular disease. Cui et al. [47] presented a case series highlighting the use of alternative arterial access routes, specifically percutaneous axillary approach, for mechanical circulatory support in patients experiencing refractory cardiac arrest. In scenarios where traditional femoral access is unfeasible due to iliofemoral arterial disease or other anatomical constraints, these alternative sites facilitated the emergent deployment of devices such as the Impella CP and ECMO cannulation. Jin et al. [36] conducted a retrospective analysis comparing femoral artery (FF) cannulation alone versus combined femoral and axillary artery (FAx) cannulation in 51 post-cardiotomy V-A ECMO patients. Despite the FAx group presenting with more preoperative risk factors, this cohort exhibited significantly lower incidences of chronic renal failure (14.81% vs. 37.50%, *p* = 0.045), higher platelet counts, and lower creatinine levels compared to the FF group. Although 30-day mortality rates were similar between two groups, the FAx approach demonstrated advantages in reducing complications and improving patient recovery. These findings suggest that incorporating axillary artery cannulation alongside femoral access may enhance hemodynamic support and mitigate complications in V-A ECMO patients. Vale et al. [39] conducted a retrospective study comparing femoro-axillary (FAx) and femoro-femoral cannulation strategies in V-A ECMO for refractory cardiogenic shock. The analysis revealed that FAx cannulation was associated with a significant reduction in complications such as limb ischemia, local infections, bowel ischemia, and pulmonary edema compared to FF cannulation. However, a higher incidence of stroke was observed in the Fax group. Importantly, 90-day mortality rates were similar between the two groups. These findings suggest that while FAx cannulation may mitigate certain complications associated with FF access, the increased risk of stroke necessitates careful patient selection and further investigation into preventive strategies.

Andrei et al. [40] conducted a prospective study evaluating the hemodynamic effects of FF vs. FAx extracorporeal life support (ECLS) configurations. Using pulse-wave Doppler to measure the velocity time integral (VTI) in the descending thoracic aorta (DTA), they observed that in FAx cannulation, DTA VTI increased proportionally with ECLS flow rates, indicating enhanced left ventricular (LV) ejection. Conversely, in FF cannulation, DTA VTI decreased as ECLS flows increased, suggesting potential LV outflow obstruction due to retrograde aortic flow. These findings highlight that FAx cannulation may better preserve native LV function and could inform strategies for ECLS weaning. Perfusion studies reveal site-specific effects. Sibut-Pinote et al. [48] evaluated the impact of arterial cannulation site (axillary vs. femoral) on coronary, cerebral, and renal perfusion during combined veno-arterial ECMO and Intra-aortic balloon pump (IABP) support under simulated low cardiac output conditions. Axillary cannulation significantly improves coronary and cerebral blood flow compared to femoral access, particularly in severe shock states. However, renal perfusion is comparatively reduced with axillary cannulation. These findings suggest that axillary cannulation may optimize cerebral and myocardial oxygen delivery during mechanical circulatory support but necessitates cautious consideration of potential renal hypoperfusion. The results support individualized cannulation strategies in cardiogenic shock management.

#### 3.3.3. Cerebral Perfusion and Neurologic Outcomes: Comparing Axillary, Femoral and Central Cannulation Strategy

A multicentric study by Chiarini et al. [41] compares neurologic complications among patients receiving post-cardiotomy ECLS via three arterial cannulation sites: aortic, subclavian/axillary, and femoral. Results show that subclavian/axillary cannulation is associated with a significantly higher incidence of major neurologic events and seizures compared to aortic cannulation. Although aortic cannulation patients exhibited high overall in-hospital mortality, neurologic causes of death did not differ significantly across groups. Instead, a retrospective study by Nishikawa et al. [42] compares stroke incidence and patterns across three arterial cannulation sites (ascending aorta, axillary artery, and femoral artery) on 414 V-A ECMO patients. Stroke rates were similar among groups (6.2–6.5%), with ischemic strokes constituting the majority (64%). The findings indicate that arterial cannulation site does not significantly impact overall stroke risk or subtype in V-A ECMO patients. Therefore, stroke prevention strategies should be uniformly applied regardless of cannulation approach. Notably, Salna et al. [43] conducted a prospective study on 37 patients undergoing peripheral V-A ECMO to assess the effects of arterial cannulation site (axillary vs. femoral) on cerebral perfusion using transcranial Doppler (TCD) ultrasonography. Axillary cannulation resulted in significantly higher middle cerebral artery flow velocities and lower pulsatility indices bilaterally, suggesting more continuous and stable cerebral flow. In contrast, femoral cannulation showed reduced cerebral flow and increased pulsatility, potentially reflecting suboptimal cerebral perfusion. Axillary artery cannulation provides superior cerebral hemodynamics compared to femoral access during V-A ECMO, highlighting the potential advantage of this site for neuroprotection in selected patients. Feiger et al. [49] employed a one-dimensional blood flow simulation model to investigate how V-A ECMO parameters influence cerebral oxygen delivery, with particular focus on cannulation site and ECMO flow rate. Axillary and central cannulation ensured adequate carotid perfusion at lower ECMO flow rates (~1 L/min), whereas femoral cannulation required substantially higher flows (>4.9 L/min) to achieve comparable cerebral perfusion. Increased ECMO flow consistently improved cerebral oxygenation across configurations, but cannulation site markedly modulated this effect. Axillary and central cannulation offer superior cerebral oxygenation efficiency compared to femoral access, especially under low-flow ECMO conditions. These findings underscore the relevance of cannulation strategy in minimizing the risk of cerebral hypoxia during V-A ECMO support.

#### 3.3.4. Complications Associated with Axillary Artery Cannulation for V-A ECMO

Complications require vigilant management. Ohira et al. [44] conducted a retrospective study comparing FAx and FF cannulation in 80 heart transplant recipients supported with peripheral V-A ECMO. The analysis revealed that FAx cannulation was associated with a significantly lower incidence of cannulation-related wound complications, including infections, compared to FF cannulation. Other outcomes, such as survival to discharge, incidence of stroke, bleeding, and limb ischemia, were comparable between the two groups. Mittal et al. [50] described two cases of brachial plexus injury associated with ECMO, highlighting axillary artery cannulation as a key risk factor. Cannulation-related hematoma formation likely contributed to nerve compression and injury. Joffre et al. [51] describe a fatal aortic dissection occurring during ECMO via axillary cannulation following cardiac arrest. The report underscores the rare but catastrophic risk of aortic dissection with axillary cannulation in ECMO. Saito et al. [52] describe massive upper-extremity edema following trans-axillary cannulation for V-A ECMO. The edema likely results from impaired venous return due to cannula-related venous obstruction or local tissue inflammation, which may lead to increased capillary permeability and fluid accumulation. Such limb swelling poses risks including compartment syndrome, tissue ischemia, and potential limb loss if not promptly addressed. Omer et al. [53] critically evaluated the use of axillary artery cannulation in V-A ECMO, highlighting its role as an alternative to femoral cannulation. Axillary access provides improved hemodynamic support and reduces risks associated with femoral cannulation, such as limb ischemia and infection. However, it is not without significant risks; it can lead to upper extremity ischemia, limb hyper perfusion, compartment syndrome, and tissue necrosis if not carefully managed. The authors stress the importance of rigorous patient selection, precise surgical technique, and vigilant postoperative surveillance to promptly identify and address vascular complications. In this context Capuano et al. [54] proposed a practical surgical technique to maintain adequate arterial flow to the upper limb during direct right axillary artery cannulation, especially in prolonged ECMO support. The method aims to prevent limb ischemia and related complications such as compartment syndrome by ensuring continuous antegrade perfusion distal to the cannulation site. They perform a side graft (usually an 8 mm8-mm Dacron graft) sewn end-to-side to the axillary artery, allowing arterial cannulation through the graft rather than directly into the artery. This preserves native arterial flow to the arm and optimizes patients’ safety by minimizing vascular compromise during ECMO, facilitating longer support duration with reduced risk of ischemic injury. Similarly, both Moazami et al. [55] and Papadopoulos et al. [56] described a technique for axillary artery cannulation designed to minimize procedure-related complications, particularly upper limb hyper perfusion, by employing a side-graft anastomosis. This approach facilitates a more physiologically balanced distribution of arterial flow.

In summary, reported complications include:

- Upper extremity ischemia, due to arterial spasm, thrombus formation or inadequate distal perfusion [35].
- Bleeding and hematoma formation, especially in anticoagulated patients or when surgical hemostasis is challenging due to anatomical constraints [50].
- Nerve injury, including brachial plexus trauma, may occur due to local hematoma or during surgical dissection [12].
- Iatrogenic pneumothorax: though uncommon, remains a procedural risk, particularly with percutaneous attempts in the infraclavicular region or in patients with emphysematous lungs, morbid obesity or altered thoracic anatomy [57].
- Limb hyper perfusion, compartment syndrome, and tissue necrosis [17].
- Cannulation performed in emergency settings, often under suboptimal conditions, may be associated with higher complications rates at the time of decannulation due to inadequate positioning, lack of vessel control or unrecognized arterial injury [58].

Axillary cannulation expands therapeutic options for complex ECLS scenarios, though its risk-benefit profile necessitates individualized decision making and technical expertise.

### 3.4. Indications for Axillary Cannulation in V-A ECMO

In the setting of V-A ECMO, axillary artery cannulation has gained increasing attention as a physiologically favorable alternative to femoral access, particularly in selected high-risk patients (Table 3).

- Anatomic indications: Axillary access is primarily indicated in individuals with significant peripheral arterial disease (PAD), morbid obesity or when preservation of upper body perfusion and cerebral oxygenation is of paramount concern. In patients with severe PAD, the iliofemoral arteries may be heavily calcified or stenotic, increasing the technical complexity and clinical risks of femoral cannulation, including limb ischemia, inadequate systemic flow, and embolic complications [59]. Similarly, in obese patients, where femoral vessels are often difficult to access and prone to infection or hemorrhagic complications due to deep subcutaneous layers, the axillary artery offers a more superficial and surgically manageable target [60].
- Physiological and Hemodynamic indications: a particularly compelling advantage of axillary cannulation in ECMO lies in its ability to deliver true antegrade systemic perfusion, thereby optimizing oxygen delivery to the cerebral and coronary circulations. This feature is especially relevant in preventing North–South syndrome (NSS) [29].
- Clinical and organizational indications: the anatomical location and stability of the axillary cannulation site offer practical advantages in the context of long-term ECMO support. Compared to femoral access, axillary cannulation is better suited for patient mobilization and active rehabilitation, which are increasingly recognized as key components of care in prolonged extracorporeal support. This further reinforces its role in advanced ECMO management, particularly in patients with expected delayed recovery or as a bridge to transplant or durable mechanical circulatory support [61].

**Figure 6:**
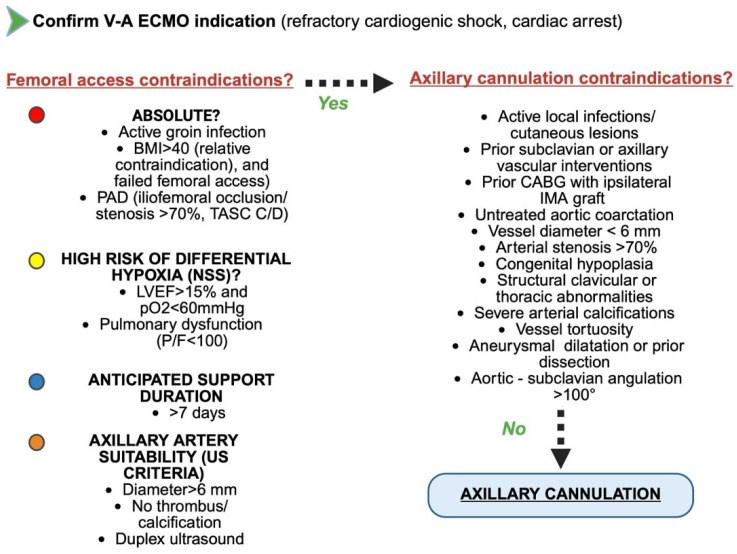
Proposed algorithm for axillary cannulation in adult requiring V-A ECMO. BMI: body mass index; PAD: peripheral artery disease; TASC: Trans-Atlantic Intersociety Consensus; NSS: North–South syndrome; LVEF: left ventricular ejection fraction; pO_2_: partial pressure of oxygen; US: ultrasonography; CABG: coronary artery bypass grafting; IMA: internal mammary artery. Created in BioRender 201.

In summary, axillary cannulation in ECMO is indicated not only in patients with anatomical barriers to femoral access, such as severe peripheral arterial disease (PAD) and morbid obesity, but also in clinical scenarios where optimized cerebral and upper body oxygenation is essential, including the prevention of North–South syndrome and the need for preserved cerebral and coronary perfusion. Furthermore, in patients with recovering left ventricular function and significant native cardiac output, axillary access promotes more favorable hemodynamic mixing dynamics and may reduce retrograde ECMO competition. Although it does not eliminate the increased afterload inherent to peripheral V-A ECMO, this approach may offer modest hemodynamic advantages, particularly when integrated with appropriate unloading strategies. Additional indications include anticipated long-term ECMO support, where axillary access is better tolerated anatomically and facilitates vascular care, as well as protocols involving early mobilization, where stable cannula positioning supports safe initiation of physiotherapy and rehabilitation. Its expanding role reflects a shift toward more individualized, physiology-guided cannulation strategies aimed at optimizing both immediate and long-term outcomes in patients with complex cardiopulmonary failure (Figure 6).

### 3.5. Contraindications, Pre-Procedural Imaging, and Limitations of Axillary Artery Cannulation

Axillary artery cannulation, while offering several advantages in extracorporeal support strategies, is not devoid of contraindications. Nevertheless, pre-procedural assessment is essential to identify anatomical and clinical factors that may hinder safe vascular access or predispose to serious complications. Patient selection must therefore be individualized and guided by multimodal imaging, including computed tomographic angiography and ultrasound, to evaluate vessel morphology, patency, and adjacent structures.

Absolute contraindications include active local infection or cutaneous lesions at the intended cannulation site, which carry a high risk of bacteremia and systemic seeding. Prior vascular interventions, such as placement of covered stents or complex surgical repairs of the axillary or subclavian arteries, may render the vessel unsuitable for percutaneous or surgical access. Furthermore, in patients with prior coronary artery bypassing grafting involving the left, the right or bilateral internal mammary arteries (LIMA, RIMA, BIMA), axillary access on the ipsilateral side poses a substantial risk of graft injury or competitive flow, potentially impairing myocardial perfusion. In such cases, contralateral cannulation is generally preferred, provided that anatomical conditions allow for it. In adult patients with uncorrected aortic coarctation, axillary artery cannulation may result in inadequate distal perfusion and increased afterload, rendering extracorporeal circulatory support hemodynamically ineffective or potentially deleterious.

Relative contraindications encompass a spectrum of morphological alterations that may complicate access or impair perfusion. A vessel diameter less than 6 mm may preclude the use of adequately sized cannulas, resulting in suboptimal perfusion pressures and increased shear stress, with a heightened risk of thromboembolic complications. Significant subclavian artery pathology, including untreated stenosis exceeding 70% or excessive atherosclerotic burden, may compromise antegrade flow, lead to ipsilateral upper extremity ischemia, and predispose to vertebral steal phenomena with cerebral hypoperfusion. Congenital hypoplasia of the axillary artery, leading to reduced luminal diameter, may compromise antegrade flow, particularly in the setting of fragile, calcified plaques that may be disrupted during cannulation. Vascular conditions such as severe arterial calcification, marked tortuosity, aneurysmal dilatation or prior dissection also increase the technical complexity of cannulation and raise the risk of procedural failure or vascular injury. Aortic-subclavian angulation greater than 100 degrees presents a particular technical challenge, potentially hampering cannula insertion, alignment, and stabilization. In addition to vascular morphology, structural alterations resulting from prior trauma or surgery, such as clavicular fixation, thoracic outlet syndrome repair or scar tissue, may distort the regional anatomy and hinder cannula placement or stability. Moreover, in patients with advanced peripheral arterial disease, axillary cannulation may compromise future vascular access options, particularly for trans-radial or trans-brachial coronary interventions or hybrid revascularization strategies, which often rely on upper extremity access [13,62,63,64,65,66].

Through pre-procedural planning, including detailed imaging assessment of vessel morphology, patency, and surrounding anatomical context, is imperative to ensure the feasibility and safety of axillary cannulation. Among the imaging modalities, CT angiography (CTA) is considered the gold standard for evaluating the axillary and subclavian arteries, allowing detailed assessment of vessel caliber, patency, tortuosity, calcifications, and anatomical variants. CTA is particularly essential in elective surgical cannulation or when there is suspicion of vascular pathology, such as stenosis, aortic arch abnormalities, grafts or previous surgeries. Duplex ultrasound is valuable for bedside evaluation of arterial diameter and flow characteristics, and it can also guide percutaneous cannulation. Its utility may be particularly relevant in urgent situations requiring rapid decision-making. Digital subtraction angiography (DSA) is rarely required before cannulation but may offer additional hemodynamic information in complex cases of peripheral arterial disease or when prior stent-grafts are present. The indications for mandatory imaging are: elective surgical axillary access, known or suspected subclavian artery disease, history of CABG with IMA grafts, suspected aortic arch abnormalities or coarctation, high risk anatomy (e.g., prior clavicular or thoracic surgery, PAD). In emergent scenarios, when immediate ECMO initiation is required, imaging may be limited to bedside ultrasound, with definitive evaluation deferred. However, this practice may increase the risk of suboptimal cannulation, resulting in malposition, distal embolism, bleeding or failure of support. Whenever feasible, even in urgency, rapid vascular assessment should be pursued [67,68,69,70,71,72].

Axillary artery cannulation is associated with several limitations that warrant careful considerations. Surgical arterial exposure is technically more challenging than femoral access, typically requiring a deltopectoral or infraclavicular approach that may prolong cannulation time, particularly in urgent or hemodynamically unstable scenarios [12]. Furthermore, the caliber of the axillary artery may restrict the use of large bore arterial cannulas, potentially limiting maximal ECMO flow rates in larger or hyperdynamic patients. In contrast to the femoral artery, which can typically accommodate 17–21 Fr cannulas allowing full-flow support (>5–6 L/min), in most cases, direct axillary cannulation supports arterial cannulas up to 15–17 Fr, enabling flow rates in the range of 3.5–4.5 L/min. This constraint becomes clinically relevant in patients with high cardiac output demands, in whom suboptimal flow may fail to achieve adequate systemic perfusion. In this type of cannulation, persistently elevated lactate levels may occasionally be observed during the first 24 h following ECMO initiation, likely reflecting suboptimal flow delivery, which typically reaches only 80% to 90% of the native cardiac output [45,73].

From a hemodynamic perspective, although axillary cannulation provides antegrade flow toward the aortic arch and supra-aortic vessels, it does not fully mitigate the increased left ventricular afterload inherent to peripheral V-A ECMO and may still necessitate adjunctive strategies for effective ventricular unloading. Another relevant drawback is that decannulation must be performed in the operating room under controlled surgical conditions. Unlike femoral access, where percutaneous or bedside removal may occasionally be feasible, axillary cannulation requires surgical repair of the artery, typically with patch angioplasty or primary closure, to prevent complications such as hemorrhage, pseudoaneurysm or arterial thrombosis. The proximity to the brachial plexus and the confined anatomical space further necessitates precise vascular control to minimize the risk of nerve injury or incomplete hemostasis [62,74].

### 3.6. Risk of Decannulation Related Complications in V-A ECMO: Axillary vs. Femoral Artery Access

Despite the growing body of literature evaluating cannulation strategies for V-A ECMO, few studies specifically focus on the arterial decannulation, and the mostly are inherently femoral access decannulation. Nonetheless, several insights can be drawn from available data and institutional practice. Femoral decannulation can be performed either surgically with direct vessel repair or percutaneously using vascular closure devices such as MANTA or Perclose ProGlide [75,76,77]. Complications include: access site bleeding, hematoma, pseudoaneurysm. Recent data suggest that percutaneous closure devices may reduce complications rate [78,79,80]. In a comparative study by Chandel et al. [81], MANTA-based decannulation was associated with fewer vascular complications than surgical closure. The axillary artery decannulation is typically performed in the operating room, due to the non-compressible nature of the artery, the proximity to the brachial plexus, the need for surgical graft revision or vessel repair. Reported complications include: hemorrhage at the graft side, neurological injury (e.g., brachial plexus involvement), limb ischemia (due to distal embolism). Importantly, ECMO cannulation is frequently performed in emergency settings, under suboptimal anatomical or hemodynamic conditions. In such scenarios, priority is rightly given to life-saving circulatory support, often at the expense of an ideal vascular access site selection or technique. As a consequence, suboptimal cannulation, such as malpositioned or oversized cannulas, insufficient distal perfusion or excessive vessel trauma, can significantly increase the risk of complications during and after decannulation, regardless of the access route. These complications may include bleeding due to vascular friability or pseudoaneurysm formation, difficult vascular repair, particularly in previously traumatized or infected tissue, and delayed wound healing or lymphocele formation. This is particularly relevant for femoral access, where rapid percutaneous cannulation without ultrasound-guidance remains common in cardiac arrest or profound shock. However, axillary access too, when emergently performed, can be complicated by non-ideal graft positioning or arterial dissection, which may render the decannulation phase technically demanding and risk-prone [74,82,83].

### 3.7. ECMELLA Configuration

In patients with severe cardiogenic shock, a combined femoro-axillary V-A ECMO strategy in conjunction with Impella support (ECMELLA or ECPELLA configuration), via either femoral or axillary access can provide both adequate systemic perfusion and effective left ventricular unloading. This combined approach helps prevent left ventricular distension, pulmonary congestion and elevated left ventricular end-diastolic pressure. The Impella device may be implanted either percutaneously via femoral artery (Impella CP/2.5) or surgically via an axillary artery graft (Impella 5.0/5.5). Axillary Impella implantation is associated with higher achievable flow rates, fewer device-related complications and the potential for early patient mobilization [84,85,86]. When axillary artery access is selected, the Impella device can be placed in the contralateral axillary artery to avoid vascular crowding. However, techniques have been described whereby both the Impella and the arterial ECMO return cannula can be implanted via the same axillary artery, provided the vessel has a minimum diameter of approximately 7 mm. In these cases, a Y-shaped graft is surgically anastomosed end-to-side to the axillary artery: one limb carries the Impella 5.0/5.5 catheter and the other limb accommodates the arterial cannula for V-A ECMO. This configuration allows simultaneous antegrade ECMO support and LV unloading through a single arterial access while minimizing additional cannulation and preserving patient mobility [87,88]. To ensure adequate perfusion and reduce the risk of inflow resistance the axillary artery must have a sufficient diameter, ideally greater than 7 mm, to allow for an extended arteriotomy and accommodate both the ECMO return cannula and the Impella catheter. In smaller caliber arteries, the presence of the Impella shaft within the arteriotomy site may significantly limit the effective lumen, potentially compromising ECMO flow [89]. One of the key advantages of axillary placement of an Impella 5.0 or 5.5 lies in its dual utility: initially as a left ventricular unloading strategy during the acute phase of severe cardiogenic shock and subsequently as a diagnostic tool to assess right ventricular (RV) function during recovery. The presence of ECMO, by mechanically unloading the RV, can obscure native RV function and hinder accurate prediction of post-left ventricular assist device (LVAD) RV failure [90]. In a large multicenter retrospective study of patients bridged to LVAD with ECMO, the need for subsequent right-sided support can be as high as 45% [91]. Axillary Impella placement allows for staged weaning, initial LV unloading during ECMO, followed by RV function assessment post ECMO in LVAD-like conditions, with reported feasibility, safety, high 1-year survival ~ 90%, and low post LVAD RV failure (~11.1%) [92]. Finally, in patients with temporary contraindications to LVAD, Impella provides an effective bridge-to-bridge or bridge to decision approach, enabling tailored hemodynamic support and informed long-term planning [93].

## 4. Discussion

The axillary artery has increasingly gained attention as a valid and potentially superior alternative to femoral artery access for V-A ECMO, particularly in patients with cardiogenic shock and severe pulmonary compromise. Its appeal lies in the ability to achieve antegrade perfusion of the aortic arch and its branches, thereby potentially improving cerebral and myocardial oxygen delivery, while avoiding many of the limitations and complications associated with femoral cannulation [18,94]. One of the principal motivations for favoring axillary artery cannulation is the mitigation of differential hypoxemia. Unlike femoral access, axillary cannulation delivers a more physiological antegrade flow, effectively perfusing the innominate and carotid arteries. This ensures more consistent cerebral oxygenation and lowers the risk of hypoxic brain injury [18,94,95]. Additionally, the axillary approach avoids the complications frequently associated with femoral artery cannulation, such as lower limb ischemia [96,97,98], vascular dissection [99], retroperitoneal hemorrhage [100,101,102], and groin infection [103]. These complications can have significant consequences, particularly in critically ill patients, and often require invasive interventions such as fasciotomy, limb revascularization or even amputation [104,105,106]. Furthermore, in patients who are expected to be mobilized during ECMO (e.g., as a bridge to transplant or bridge to recovery candidates), axillary access allows for greater patient mobility and potentially reduces ICU-acquired complications such as muscle wasting and delirium [107]. Nevertheless, despite its physiological and theoretical advantages, axillary artery cannulation is not devoid of drawbacks. It is technically more demanding, often requiring surgical exposure and graft interposition (typically a side graft anastomosed to the artery) to minimize the risk of arterial injury and thrombosis [9,18,19]. The procedure is time-consuming and operator-dependent, and not all centers possess the surgical expertise or resources to perform it rapidly in emergency settings. Furthermore, complications such as bleeding, seroma formation, graft infection, brachial plexus injury, and thromboembolism have been reported, albeit at variable incidence rates across the literature [50,51,52,53,54,55]. Although several studies have demonstrated favorable outcomes [12,31,43,49], including reduced rates of neurological complications, limb ischemia, and North–South syndrome, these findings are not universally consistent. Some studies report [42,47] comparable mortality and complication rates between axillary and femoral cannulation, suggesting that outcomes may depend more on patient-specific factors and center expertise than on cannulation site alone. A limitation of the existing literature is the lack of standardized criteria for selecting patients for axillary versus femoral cannulation. Variables such as aortic arch anatomy, degree of pulmonary dysfunction, anticipated ECMO duration, presence of peripheral vascular disease, and timing of cannulation (emergent vs. elective) are inconsistently reported and rarely stratified in outcome analyses. This heterogeneity complicates the ability to draw definitive conclusions for cannulation strategy. Emerging approaches, such as percutaneous axillary cannulation under ultrasound or fluoroscopic guidance [21], offer the potential to simplify the procedure and reduce surgical morbidity. However, data on their safety and efficacy are currently limited and largely anecdotal. Moreover, the feasibility of such techniques in unstable or coagulopathic patients remains questionable and warrants further investigation.

Looking forward, there is a pressing need for well-designed, multicenter, prospective studies to rigorously compare axillary and femoral cannulation in terms of mortality, end organ perfusion, neurological outcomes, infection rates, and long-term functional recovery. Randomized controlled trials would be ideal, but logistical and ethical challenges in the ECMO population may render them difficult to conduct. In the interim, large scale registry data and propensity-matched analyses could provide valuable insight and help refine patient selection criteria.

In summary, a proposed algorithm for V-A ECMO implantation in the setting of cardiac arrest or refractory cardiogenic shock should begin with an evaluation of contraindications to femoral cannulation. These contraindications include a body mass index > 40 (relative contraindication), peripheral arterial disease classified as Trans-Atlantic Intersociety Consensus (TASC) II C/D, with iliofemoral occlusion or stenosis > 70%, and active groin infection [107]. If any of these contraindications are present, axillary artery cannulation should be considered.

In patients with extreme obesity, femoral cannulation is technically challenging and associated with significantly higher rates of vascular complications, including hematoma, limb ischemia, infection, and cannula malposition due to the depth of the vessel and the overlying adipose tissue. Furthermore, ultrasound-guided access becomes more technically demanding [108,109]. Axillary cannulation, particularly via a surgical cutdown approach, provides a more stable and superficial access point with improved hemostatic control, allowing for safer cannulation and more reliable upper body perfusion in high-risk cohort.

The presence of a local infectious process in the inguinal region is an absolute contraindication to femoral arterial cannulation due to the heightened risk of retrograde infection, bacteremia, and cannula contamination [107]. In such cases, the axillary artery, which is anatomically remote from the infected field, represents a safer and sterile alternative. Its cannulation avoids exposure of ECMO circuit to purulent contamination and mitigates the risk of systemic sepsis.

In patients with advanced aorto-iliac occlusive disease (TASC II types C or D) and hemodynamically significant (>70%) stenosis or occlusion of the iliac or femoral arteries, femoral access not only poses high technical failure rates but also renders distal perfusion inadequate [34,110]. Retrograde arterial flow from a femoral cannula may fail to overcome proximal obstruction, exacerbating ischemia in the lower extremities and failing to deliver adequate cardiac support. In contrast, axillary cannulation allows antegrade perfusion of the aortic arch and cerebral vessels and is thus preferred in the setting of extensive peripheral arterial disease.

Moreover, axillary cannulation should also be contemplated if the patient is suspected to be at risk of developing North–South syndrome (e.g., left ventricular ejection fraction > 15% with concomitant respiratory failure) or in scenarios where long-term mechanical circulatory support (MCS) is anticipated, either as a bridge to transplantation or durable MCS [20]. Concurrently, the suitability of the axillary artery for cannulation must be carefully assessed.

Prior to axillary cannulation duplex ultrasonography and CT angiography is recommended to assess vessel diameter, wall integrity, and flow characteristics, ensuring suitability for cannulation and minimizing the risk of distal ischemia or dissection [111]. The axillary artery must have a minimum luminal diameter of 6 mm to safely accommodate standard ECMO arterial cannulas up to 17 Fr (approximately 5.67 mm in outer diameter), allowing for an adequate safety margin to prevent vascular injury and preserve distal perfusion. Smaller calibers are associated with higher shear stress, turbulent flow, and increased likelihood of cannula-induced arterial injury. Nonetheless, specific patient conditions may limit the feasibility or safety of axillary access. Subclavian artery disease, previous stenting or unrepaired aortic coarctation, axillary hypoplasia, can impair antegrade flow or exacerbate cerebral and systemic malperfusion. Prior use of internal mammary artery grafts, particularly when originating from the subclavian or proximal axillary artery, may be vulnerable to competitive flow or injury during cannulation. Moreover, thoracic outlet abnormalities, aneurysm and advanced peripheral arterial disease may compromise access or limit future revascularization strategies requiring upper-extremity routes. Importantly, active infection at the cannulation site constitutes an absolute contraindication to axillary artery cannulation [66].

Several important limitations should be acknowledged when interpreting both the current literature and the conclusion of this narrative review. First, the majority of available studies examining axillary artery cannulation in V-A ECMO are retrospective analyses or single-center case series. Randomized controlled trials (RCTs) and high-quality meta-analyses are exceedingly rare. This predominance of lower-level evidence increases the risk of selection bias, as patients undergoing axillary cannulation are often selected based on clinical stability or institutional preference, potentially skewing the outcomes. Consequently, the ability to generalize findings across broader patient populations is limited. Second, there is a considerable variability in the techniques used, ranging from open surgical side graft to fully percutaneous ultrasound or fluoroscopy-guided access. These differences reflect institutional expertise and availability of resources, but they also introduce procedural heterogeneity that complicates the interpretation of results across studies. Third, the impact of axillary cannulation on neurological outcomes remains controversial. Some studies, such as that Chiarini et al. [41], have reported an increased incidence of major neurologic events and seizures in patients with subclavian or axillary cannulation. In contrast, Nishikawa et al. [42], found no significant differences in stroke incidence or patterns among patients cannulated via the ascending aorta, femoral artery or axillary artery. These conflicting results may be attributable to various confounding factors, including anticoagulation regimens, ECMO flow rates, underlying patient comorbidities, and the use (or lack) of cerebral monitoring. Such discrepancies underscore the importance of interpreting neurologic outcomes with caution and of developing strategies to mitigate risk, such as standardized heparinization protocols and real-time neuromonitoring using near-infrared spectroscopy (NIRS) or transcranial Doppler. Another important consideration concerns the conflicting data regarding end organ perfusion. While axillary cannulation appears to offer superior cerebral and coronary oxygenation, particularly in the context of North–South syndrome, it may compromise perfusion to other organs, such as the kidneys. As demonstrated by Sibut-Pinote et al. [48], renal blood flow was comparatively reduced with axillary access, likely due to the redistribution of ECMO output toward the upper body. These findings, highlights the need to balance the physiological advantages of antegrade cerebral perfusion with the risk of visceral hypoperfusion, tailoring the cannulation strategy to each patient’s clinical priorities and organ vulnerabilities.

In conclusion, axillary artery cannulation represents a promising alternative to femoral access in selected V-A ECMO patients, offering distinct physiological advantages and potentially improved clinical outcomes. However, its implementation should be tailored to the patient’s clinical context and institutional expertise. Until more definitive evidence becomes available, the choice of cannulation strategy should remain individualized, guided by a comprehensive assessment of risk and benefit.

## 5. Conclusions

Axillary cannulation has emerged as a compelling alternative to femoral access in selected patients undergoing V-A ECMO, particularly in the context of contraindications to femoral cannulation or increased North–South syndrome risk. Its capacity to deliver antegrade perfusion to the aortic arch and cerebral circulation offers a physiologically advantageous route that may mitigate differential hypoxemia and reduce the incidence of neurologic injury and lower limb ischemia. Moreover, its utility in facilitating patient mobilization renders it especially attractive in bridge-to-transplant or bridge-to-recovery strategies. However, axillary access is not without limitations. Its technical complexity, the need for surgical expertise, and the variability in procedural approaches and patient selection criteria across institutions limit the generalizability of current evidence. Additionally, the potential for complications such as brachial plexus injury, vascular trauma, and visceral hypoperfusion underscores the necessity for rigorous patient selection and vigilant perioperative management. The paucity of high-quality prospective data and randomized controlled trials continues to hinder definitive conclusions regarding its superiority over femoral access. Future investigations should prioritize multicenter, prospective, and methodologically robust studies aimed at stratifying patients according to anatomical, hemodynamic, and respiratory parameters to better guide cannulation strategy. Integration of preoperative vascular imaging, cerebral and end-organ perfusion monitoring, and standardized anticoagulation protocols will be critical to optimizing outcomes. Until such evidence becomes available, axillary cannulation should be regarded as a valuable yet context-dependent modality, best deployed within experienced centers and tailored to the individual patient’s anatomical and clinical profile.

## Figures and Tables

**Table 1:** Summary of clinical studies (retrospective and prospective) for Axillary arterial cannulation in Extracorporeal life support. These studies represent the current higher-level evidence available in this field, though still limited, in simple size and design heterogeneity. FAx: femoro-axillary ECMO; FF: femoro-femoral ECMO; LV: left ventricle; IABP: intra-aortic balloon pump; MCA: middle cerebral artery; ECLS: extracorporeal life support; ECPR: extracorporeal cardiopulmonary resuscitation; VTI: velocity time integral (aortic).

Study	Design	Population	Key Findings	Complications
Ohira et al. (2020) [12]	Retrospective	Cardiogenic shock patients on V-A ECMO (*n* = 371)	Axillary access reduced limb ischemia, wound complications, and site conversion vs. femoral access, with equivalent survival and no increased bleeding or stroke	↓ limb ischemia vs. femoral cohort
Chamogeorgakis et al. (2013) [17]	Retrospective	Adult V-A ECMO patients (*n* = 81 axillary side-graft, 166 femoral, 61 aortic)	Axillary artery cannulation using a side graft was associated with safe and effective extracorporeal support, facilitated patient mobilization, and significantly reduced lower limb ischemia compared to femoral access	Ipsilateral upper limb hyperperfusion syndrome (24.7%), graft site bleeding (17.3%). Femoral access was associated with higher rates of lower limb ischemia and fasciotomy
Cakici et al. (2017) [19]	Retrospective observational cohort	V-A ECMO (percutaneous vs. side graft) (*n* = 148)	Side-graft technique had fewer perfusion related complications and improved limb perfusion vs. percutaneous access	Acute limb ischemia (2.7% side-graft vs. 5.3% percutaneous), bleeding (12% side graft vs. 24.7% percutaneous), hyperperfusion syndrome (2.7% percutaneous vs. 30% side-graft). Survival outcomes were similar between groups
Liu et al. (2025) [21]	Prospective observational	ECPR patients with US-guided percutaneous axillary access (*n* = 7)	US-guided axillary access was feasible	US-guided percutaneous axillary cannulation is feasible
Radwan et al. (2023) [35]	Retrospective	Post-cardiotomy V-A ECMO via right axillary artery (*n* = 179)	FAx weaning success: 48.6%; in-hospital survival: 34.6%; 1-year survival: 74% (among weaned)	Subclavian bleeding (13.4%), upper limb ischemia (6.1%), stroke (10.6%), intracerebral hemorrhage (5%)
Jin et al. (2024) [36]	Retrospective	Post-cardiotomy V-A ECMO FF vs. Fax (*n* = 51)	FAx group: ↓ chronic renal failure (14.81% vs. 37.50%), ↑ platelets, ↓ creatinine vs. FF. Similar 30-day mortality	FAx reduced renal/metabolic complications
Pisani et al. (2021) [37]	Observational (*n* = 174)	V-A ECMO via right axillary artery (*n* = 174)	FAx feasible approach; 1-year survival: 72.7% (weaned patients)	Bleeding (4%), upper limb ischemia (1.1%), local infection (1.7%), brachial plexus injury (0.6%)
Hysi et al. (2013) [38]	Case series	ECMO requiring direct axillary cannulation (*n* = 16)	Axillary direct cannulation (no graft) provided reliable perfusion with low neurovascular complications	Minimal neurologic/vascular complications
Vale et al. (2024) [39]	Retrospective	Refractory cardiogenic shock: FAx vs. FF cannulation (*n* = 534)	FAx ↓ limb ischemia, local infections, bowel ischemia, pulmonary edema vs. FF. Similar 90-day mortality	↑ Stroke in FAx group
Andrei et al. (2019) [40]	Prospective	FF vs. FAx ECLS configurations (*n* = 11)	FAx: ↑ LV ejection (↑ VTI in descending aorta with > ECMO flow). FF: ↓ VTI (LV outflow obstruction from retrograde flow)	Hemodynamic evidence of LV compromise with FF ECMO
Chiarini et al. (2024) [41]	Multicentric	Post-cardiotomy ECLS: aortic vs. axillary vs. femoral (*n* = 1897)	FAx: ↑ major neurologic events/seizures vs. aortic	Highest neurologic risk with axillary;
Nishikawa et al. (2021) [42]	Retrospective	V-A ECMO aortic vs. axillary vs. femoral (*n* = 414)	Stroke rates similar (6.2–6.5%); ischemic strokes (64%) across territories.	Uniform risk regardless of cannulation site
Salna et al. (2019) [43]	Prospective	Peripheral V-A ECMO: axillary vs. Femoral (*n* = 37)	FAx: ↑ MCA flow velocity, ↓ pulsatility index, ↑ cerebral perfusion stability. FF: ↑ pulsatility, suboptimal perfusion	Axillary optimizes cerebral hemodynamics
Ohira et al. (2022) [44]	Retrospective	Heart transplant recipient on V-A ECMO FAx vs. FF (*n* = 80)	FAx ↓ cannulation related wound infections vs. FF. Survival, stroke, bleeding, limb ischemia equivalent	Site-specific infection advantage with FAx
Rastan et al. (2010) [45]	Retrospective	Post cardiotomy cardiogenic shock patients on V-A ECMO. (*n* = 517) 60.8% central cannulation 39.2% peripheral cannulatio (30.3% Fax ECMO)	Peripheral cannulation, including Fax experienced suboptimal ECMO flow (80–90% of cardiac output) compared with central cannulation	Cerebrovascular events (17.4%), gastrointestinal bleeding (18.8%), renal failure requiring dialysis (65%). Comparable survival and complication rates between central and peripheral access

**Table 2:** Summary of case reports, technical notes, and computational models. These studies are characterized by low statistical power but provide valuable insights in a field where high-level evidence is currently lacking. Fax ECMO: femoro-axillary Extracorporeal membrane oxygenation; FF ECMO: femoro-femoral ECMO; ECLS: extracorporeal life support.

Study	Design	Population	Key Findings	Complications
Ahmed et al. (2020) [8]	Case report	Patient on V-A ECMO with cardiogenic shock with axillary access (*n* = 1)	Successful use of Axillary artery cannulation for V-A ECMO using a side graft approach. The technique allowed early ambulation and reduce the risk of lib ischemia.	No cannulation-related complications reported
Navia et al. (2005) [46]	Technical report	Not specified	Describes the utilization of right axillary artery cannulation for ECMO support, emphasizing the technique’s feasibility in preserving cerebral and upper body perfusion while minimizing limb ischemia	Not reported
Cui et al. (2019) [47]	Case series (*n* = 3)	Refractory cardiac arrest with percutaneous axillary access	The axillary artery provided a feasible and effective alternative access route for V-A ECMO initiation in the setting of cardiac arrest and refractory shock, especially when femoral access was contraindicated or technical challenging	No access-related complications
Sibut-Pinote et al. (2025) [48]	Perfusion simulation	Combined ECMO-IABP in low cardiac output	FAx ↑ coronary/cerebral perfusion vs. FF; ↓ renal perfusion	Site-dependent perfusion trade-offs (renal hypoperfusion with axillary)
Feiger et al. (2020) [49]	Computational model	Simulated V-A ECMO flows	Axillary/Central sites: adequate carotid perfusion at 1 L/min. Femoral: required > 4.9 L/min for equivalent perfusion	Potential implications for cerebral hypoperfusion or hyperperfusion depending on ECMO flow and cannulation strategy
Mittal et al. (2013) [50]	Case report (*n* = 2)	ECMO patients	Axillary cannulation linked to brachial plexus injury from hematoma-induced compression	Neurologic injury due to local hematoma
Joffre et al. (2017) [51]	Case report (*n* = 1)	ECMO via axillary cannulation	Fatal aortic dissection during cannulation	Catastrophic vascular injury
Saito et al. (2023) [52]	Case report (*n* = 1)	V-A ECMO via trans-axillary cannulation	Massive upper extremity edema from venous obstruction/inflammation	Massive upper extremity edema
Omer et al. (2020) [53]	Expert commentary	*-*	Highlights the limb-sparing and cerebral perfusion benefits of axillary cannulation.	*-*
Capuano et al. (2011) [54]	Technique description	*-*	Side-graft anastomosis (e.g., 8 mm8-mm Dacron) preserved antegrade limb flow, ↓ ischemia. Emphasizes the importance of graft orientation and anastomotic configuration to prevent upper limb hyperperfusion	Mitigated upper limb ischemia/compartment syndrome
Moazami et al. (2003) [55]	Technique description	*-*	Describes a surgical approach for axillary artery cannulation aimed at reducing access-related complication during ECMO support. Highlights the importance of graft tunneling and secure fixation to prevent limb ischemia	No significant complications reported; the technique was developed to minimize risk of bleeding and neurovascular injury.
Papadopoulos et al. (2012) [56]	Technique description	*-*	Side-graft anastomosis reduced hyperperfusion complications via balanced flow distribution	Optimized hemodynamic profile

**Table 3:** Indications and physiological advantages of axillary cannulation in V-A ECMO. PAD: peripheral arterial disease; LV: left ventricle.

Indication/Clinical Scenario	Rationale/Benefit
Severe femoral PAD	Avoids diseased femoral vessels; ensures reliable arterial inflow
Morbid obesity	Facilitates surgical access; reduces risk of infection and hemorrhagic complications
Risk of North–South syndrome	Provides antegrade perfusion to aortic arch; preserves cerebral and coronary perfusion
Requirement for preserved cerebral perfusion	Improves upper body oxygen delivery
Native cardiac output with recovering LV function	Promotes favorable mixing dynamics; limits excessive retrograde ECMO competition
Risk of increased afterload and impaired LV unloading	May offer modest hemodynamic benefits but does not eliminate the need for additional LV unloading strategies in patients with impaired ventricular function, as peripheral V-A ECMO intrinsically increases afterload
Anticipated long-term ECMO support	Better tolerated anatomically; allows improved patient management and access for vascular care
Early mobilization strategy	Provides stable cannula positioning; facilitates active physiotherapy and rehabilitation in selected cases

## Data Availability

No new data were created or analyzed in this study. Data sharing is not applicable to this article.

## Abbreviations

The following abbreviations are used in this manuscript:

ECMO	Extracorporeal membrane oxygenation
LV	Left ventricle
FF	Femoro-femoral
FAx	Femoro-axillary
V-A	Veno-arterial
NSS	North–South syndrome
LVEF	Left ventricular ejection fraction
PAD	Peripheral artery disease
BMI	Body mass index
